# New West Palaearctic Species of *Empis chioptera* Group (Diptera: Empididae)

**DOI:** 10.3390/insects16111177

**Published:** 2025-11-18

**Authors:** Miroslav Barták

**Affiliations:** Department of Zoology and Fisheries, Faculty of Agrobiology, Food and Natural Resources, Czech University of Life Sciences Prague, Kamýcká 129, 165 00 Praha-Suchdol, Czech Republic; bartak@af.czu.cz

**Keywords:** descriptions, redescriptions, taxonomy, distribution, Europe

## Abstract

The following six new species are described and illustrated: *Empis* (*Empis*) *lusitanica* sp. nov. (Portugal), *E.* (*Empis*) *manteigasensis* sp. nov. (Portugal, Spain), *E.* (*Empis*) *miroslavi* sp. nov. (Portugal), *E.* (*Empis*) *moceki* sp. nov. (Bulgaria), *E.* (*Empis*) *serviae* sp. nov. (Spain), and *E.* (*Empis*) *troyanensis* sp. nov. (Bulgaria, Slovakia, and Italy). Diagnostic characters are discussed. The key to all West Palaearctic species of *E.* (*Empis*) *chioptera* group is provided.

## Simple Summary

This paper aims to contribute to the knowledge of Diptera in the West Palaearctic region. An understanding of individual species is a prerequisite for estimating their functional value with regard to the ecosystem services they provide. In light of the unprecedented reduction in biodiversity and the threat of further mass extinctions, we must preserve specimens in collections for future research. The genus *Empis* (Empididae, Empidinae) sensu lato is a large genus, comprising approximately 1000 described species worldwide. Here, we provide descriptions of six species new to science from Portugal, Spain, Bulgaria, Slovakia, Italy, Switzerland, Romania, Austria, France, and Czechia. Characters important for morphological identification are discussed and illustrated, and an identification key to all West Palaearctic species of the *Empis chioptera* group is provided.

## 1. Introduction

In light of the unprecedented reduction in biodiversity and the possible further mass extinction of biota, we should save species at least in collections for further studies. The present paper aims to add some knowledge concerning the Diptera of the West Palaearctic. The genus *Empis* Linnaeus, 1758 (Empididae, Empidinae), s. lato is a large genus comprising about 1000 described world species ([1] and more recently described species in numerous papers, e.g., [2,3,4,5,6,7,8,9,10,11]). Chvála and Pont [4] discussed in detail the problems of taxonomy of *Empis* and *Rhamphomyia*, which are undoubtedly paraphyletic genera. Many species and species groups thus remain unplaced in their subgenera [12]. Especially Gondwanan species and genera have for a long time compromised the traditional conception of Empidinae or new genera are erected [13]. For more information about the systematics of the tribe Empidini, see [14].

One worldwide, highly diversified, and speciose lineage is often treated as one nominal group, *Empis* (*Empis*). The Palaearctic fauna of the nominotypical subgenus *Empis* currently includes about 170 species [10], with about 150 species from Europe [5]. According to Kustov & Shamshev [7] and Daugeron et al. [15], at the very least, West Palaearctic species are characterised as male by the holoptic head; proboscis with strongly sclerotised labium, labella being very slender without pseudotracheae; antenna with a short scape and pedicel; acrostichals being present; radial fork being broadly opened; and hypandrium being well sclerotised. Females are often characterised by pennate legs.

*Empis* (*E*.) *chioptera* group may be characterised (within nominotypical subgenus) by small to very small black (mostly 2–4 mm, rarely up to 5.5 mm) species with black legs. Haltere black or yellowish brown (*E. dasyprocta* Loew, 1867), but never clear yellow. Vein M_1_ complete, whereas R_4+5_ forked. Mesoscutum without conspicuous stripes. Abdominal setae black to yellow. Prosternum without setae. Male hypopygium small, phallus short (sometimes not visible from outside except tip or base), without loops or sudden bends, merging epandrial lamellae or cerci from below. Most species with cercus larger than epandrium (except *E. aestiva* complex—in this case anal vein incomplete and abdominal setae black). Male 8th tergite without outgrowths. Females without pennate setae on mid coxa.

The *Empis* (*E*.) *chioptera* group is formally defined within the Palearctic species of the subgenus *Empis* (*Empis*). Within this group, we may recognise two complexes of closely related species, the *Empis chioptera* complex (with cercus high and L-shaped, without spines medially) and the *Empis aestiva* complex (with cercus much narrower than epandrium and with spines oriented medially). In addition, the group contains species that cannot be classified into either complex (*Empis moceki* sp. nov.).

## 2. Materials and Methods

The material studied originated from a collection from the Czech University of Life Sciences, Prague (CULSP), and partly from the National Museum, Prague. The material stored in CULSP was collected by means of mass trapping methods (sweeping vegetation, Malaise traps) and stored in 70% ethyl alcohol. Voucher specimens were selected and dried from ethyl alcohol using the method described in [16].

Genitalia preparations and drawings: Genitalia, together with the preceding 2–3 abdominal segments, were removed from the rest of the body using small scissors and macerated in potassium hydroxide solution (approx. 10%) in small vials submerged in hot water for 1–2 h. After neutralising with 8% acetic acid (5 min), the genitalia were dissected in glycerine and photographed. The photos were produced using a Nikon SMZ 1500 stereomicroscope equipped with a Canon EOS 700D digital camera. Images served as models for hand drawings, and details were added by directly observing the dissected genitalia.

The morphological terms used here follow [17,18,19]. All body measurements (including body and setae length) were taken from dry specimens (therefore, the actual length may differ from that of fresh or wet-preserved material) by means of an ocular micrometre mounted on a Nikon SMZ 1500 binocular microscope. Male body length was measured from the antennal base to the tip of the genitalia, and female body length from the antennal base to the tip of the cerci. Thoracic setae are counted on one side of the body except scutellars.

## 3. Taxonomy

**

***Descriptions of New Species***

**

***Empis* (*Empis*) *lusitanica* sp. nov.**

Zoobank link: urn:lsid:zoobank.org:act:F1DB8AA2-7D1A-42E3-A7F4-8327D503B0E1

(Figure 1A–D)

**Type material: HOLOTYPE** ♂, **Portugal**, 7 km E of Manteigas, nr. river, 580 m, sweeping, 40°24′42″ N, 7°28′4″ W, Barták M., 23.v.2008 (CULSP). **PARATYPES**: 6♂, 8♀, same data as holotype—(CULSP).

**Diagnosis:** Small species of *E*. (*Empis*) *chioptera* group (*chioptera* complex) with black body and entirely black setae. Wing milky white, anal vein incomplete. Male mid tibia with long preapical anterodorsal seta. Epandrium with long setae on apex. Female fore tibia and first tarsomere pennate dorsally, hind tibia dorsally with short pennation at least on basal half.

**Etymology:** The species is a patronym derived from the Roman province Lusitania, covering much of contemporary Portugal.

**Figure 1:**
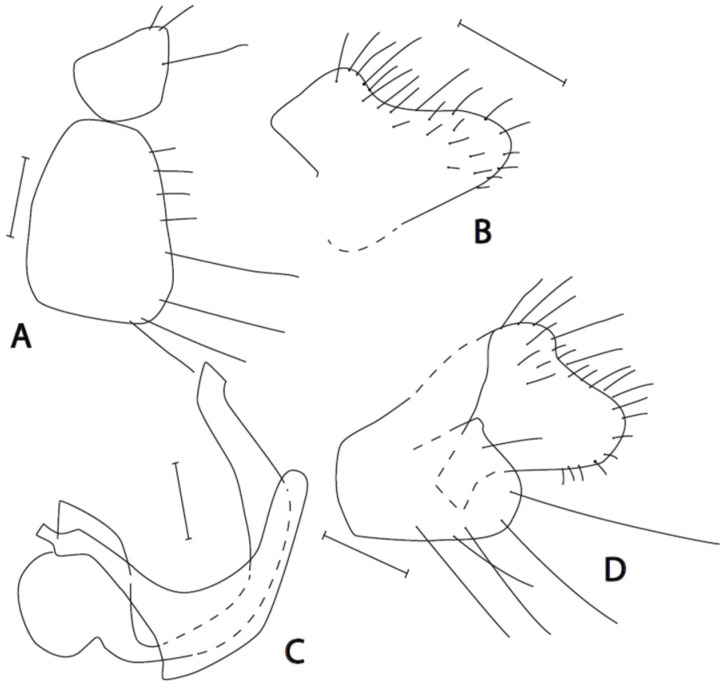
*Empis lusitanica* **sp. nov.** Postabdomen. **A** = Eighth abdominal segment; **B** = Cercus; **C** = Phallus with hypandrium; **D** = Epandrium + cercus. Paratypes. Scale lines = 0.10 mm.

**Description. Male: Head** black, dark brownish grey microtrichose, holoptic, eyes meeting over long distance. Frons (small triangles just above antennae and below front ocellus) without setae. Dorsal half of eye with larger facets than ventral half. Ocellar setae black, slightly less than half as long as frons. Occiput in dorsal third with irregularly arranged setae, postocular row irregular in lower part; dorsal postocular setae subequally long as ocellars. Face about 0.15 mm broad ventrally, microtrichose with lustrous ventral margin, without setae. Clypeus lustrous, gena very narrow, microtrichose. Palpus black, with 2–3 rather long setae basally and 1–3 slightly shorter setae apically. Labrum black, lustrous, 1.3–1.5 times longer than head height. Antenna black, both basal segments very short setose; length of antennal segments (scape: pedicel: postpedicel: basal joint of stylus: last joint of stylus, in 0.01 mm units) = 5–6: 6–7: 23–24: 2: 12–13. **Thorax** black, dark brownish grey microtrichose; mesoscutum microtrichose in dorsal view. Chaetotaxy: All setae and setulae black. Antepronotum with row of setae; proepisternum and propleura with several setae; prosternum bare; acrostichals biserial and long (about 0.15 mm, 6–8 setae in one row); dorsocentrals equally long, irregularly biserial and slightly diverging, ending in 2 longer prescutellars; 1 long postpronotal and several additional setae; 1–2 presutural supra-alar, 1–2 presutural intra-alar; notopleuron with 3 long and strong setae and with 1–3 additional setae anteriorly; 1–2 postsutural supra-alar; 2–3 pre-alars; 1 post-alar; 2 pairs of scutellars, outer pair shorter; laterotergite with black setae. **Legs** including coxae brownish black, microtrichose, entirely black setose. *Fore leg*: Femur with short dorsal and ventral setae except several preapical antero- and posteroventrals longer than femur depth; tibia with short ventral ciliation, posterodorsally with setae up to twice tibia depth; first tarsomere slightly broader than tibia, dorsally with setae about as long as tarsomere depth, ventrally with several spine-like setae longer than tarsomere depth, preapical setae strong, about one third as long as tarsomere length; ratio of tibia: first tarsomere length = 1.7–1.9; tarsomeres 2–3 with preapical setae as long as those on first tarsomere, nearly as long as these tarsomeres. *Mid leg*: Femur dorsally short setose, ventrally with two sparse rows of setae 1.5 times longer than femur depth on basal half and shorter apically; tibia with 3–4 anterodorsal setae up to three times tibia depth including long preapical seta, 2–4 posterodorsal setae shorter, 1–2 anteroventral and 5–6 posteroventral setae, at most, twice as long as tibia depth; first tarsomere very narrow, dorsally short setose, ventrally with several spines longer than tarsomere depth, preapical setae on tarsal segments slightly shorter than those on fore leg. *Hind leg*: Femur with several anteroventral or ventral setae, the longest one third before tip up to twice as long as femur depth, several anterodorsals or anterior (in middle of femur) long setae; tibia with ventral setae about as long as tibia depth, and with 0–1 slightly longer anteroventral seta, dorsally with two rows of 5–7 setae slightly longer than tibia depth; first tarsomere as wide as tibia or slightly narrower, dorsally with several longer setae similar to those on tibia, ventrally with several spines. Comb at tip of hind tibia without longer seta. **Wing** membrane milky white, veins C, R_1_, and at least apical half of R_4+5_ brown to brownish yellow, remaining veins white, axillary angle slightly acute; costal seta short; anal vein (CuA_1_) indistinct in apical third. Halter black to dark brown, calypter greyish white with darker parts, with black fringes. **Abdomen** blackish brown, brownish grey microtrichose dorsally, tergites laterally almost lustrous, most conspicuously on tergites 2–5, all setae black. Lateral setae on tergites slightly shorter than corresponding segments, marginals not differentiated. Sternites very slightly sublustrous, with medium long setae, long posteromarginals not differentiated, sternite 1 without setae. Genitalia (Figure 1A–D): cercus subequally large as epandrium, with lower lobe rounded and short setose at apex; epandrium short, sparsely long setose at apex; hypandrium long (as long as last straight part of phallus), bare and rounded at tip; phallus rather short and thick, slightly broadened submedially. **Length:** body 3.0–3.4 mm, wing 2.6–3.0 mm.

**Female:** Head dichoptic, frons about 0.15 mm wide, with several short setae on each side. Face slightly broader than frons. Thorax with setae shorter than in male. Wing slightly brownish, veins yellowish brown. *Fore leg*: Femur very short setose, dorsal setae on basal part with tendency to be slightly flattened; tibia and first tarsomere with short pennation dorsally. *Mid leg*: Femur slightly flattened, dorsally with short dense pennation, ventrally almost bare except several flattened setae preapically; tibia slightly broadened, dorsally with setae slightly flattened. *Hind leg*: Femur flattened, setosity as mid femur; tibia dorsally with short pennation at least on basal half, ventrally almost bare. Tarsi of all legs unremarkable. Abdomen with tergites 2–3 sublustrous, remaining parts microtrichose. Setae very short, sternites almost bare. **Length:** body 2.6–3.1 mm, wing 2.5–3.1 mm.

**Remarks:** *Empis* (*Empis*) *lusitanica* sp. nov. is a member of the *E*. (*E*.) *chioptera* group, *E. chioptera* complex. The most allied species is *E. pusio* Egger, 1860; however, both species differ in the shape of the genitalia. Epandrium in *E. lusitanica* sp. nov. is subequally as long as the cercus, apically with several long setae; in *E. pusio*, the epandrium is elongate, longer than the cercus, with only short setulae around the tip. Moreover, the lower lobe of the cercus is narrowed and rounded apically but broadened in *E. pusio* (compare Figure 1 with figs 11 F and G in [20]).

**

**

***Empis* (*Empis*) *manteigasensis* sp. nov.**

Zoobank link: urn:lsid:zoobank.org:act:3275B945-596B-4DB0-9899-6437ECCFA7AF

(Figure 2A–E)

**Type material: HOLOTYPE** ♂, **Portugal**, 7 km E of Manteigas, nr. river, 580 m, sweeping, 40°24′42″ N, 7°28′4″ W, Barták M., 23.v.2008 (CULSP). **PARATYPES**: 2♂, **Spain**, Pr. Sevilla, Aznalcollar, 100 m, 16.iv.1980, leg. W. Schacht—(NMP).

**Diagnosis:** Small species of *E*. (*Empis*) *chioptera* group (*aestiva* complex) with black body and brown to black setae. Fore tibia with very short posterodorsal setae but conspicuous anterodorsals. Mid tibia and first mid tarsomere with long preapical anterodorsal seta. Hind tibia slightly broadened and flattened. Anal vein incomplete, wing stigma brown. Phallus broadened in basal two thirds and very narrow apically. Female unknown.

**Etymology:** The species name is a patronym derived from holotype locality.

**Figure 2:**
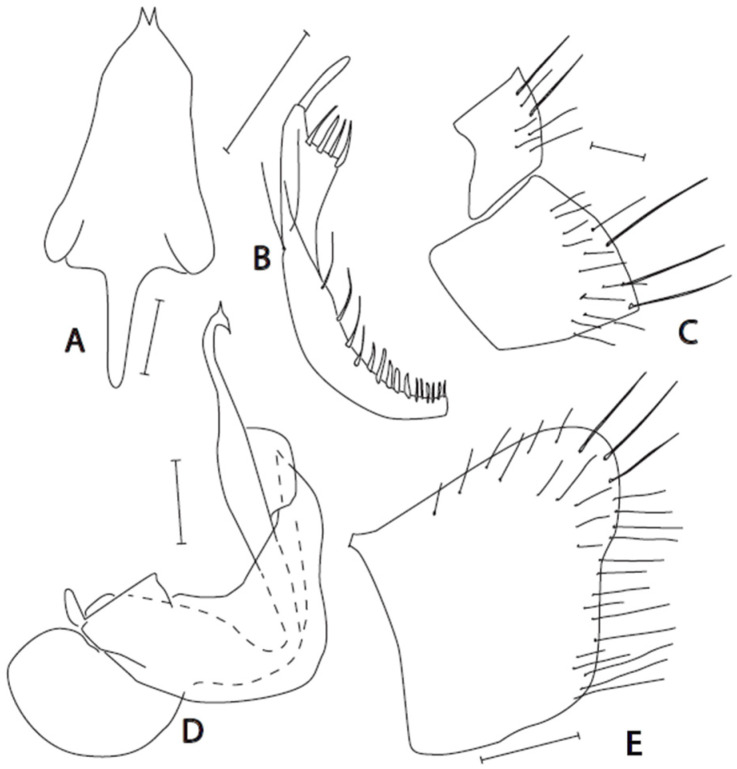
*Empis manteigasensis* **sp. nov.** Postabdomen. **A** = Hypandrium, ventral view; **B** = Cercus, dorsal view; **C** = Eighth abdominal segment, **D** = Phallus with hypandrium; E = Epandrium. Paratypes. Scale lines = 0.10 mm.

**Description. Male: Head** black, dark grey microtrichose, holoptic, eyes meeting over long distance. Frons (small triangles just above antennae and below front ocellus) without setae. Dorsal half of eye with larger facets than ventral half. Ocellar setae black, slightly less than half as long as frons. Occiput in dorsal third with irregularly arranged setae subequally long as ocellars, postocular row irregular in lower part. Face about 0.15 mm broad ventrally, microtrichose, with lustrous ventral margin, without setae. Clypeus microtrichose, gena very narrow, microtrichose. Palpus black, with about 8 rather long setae distributed nearly over its length. Labrum black, lustrous, 1.3–1.6 times longer than head height. Antenna black, both basal segments short setose; length of antennal segments (scape: pedicel: postpedicel: basal joint of stylus: last joint of stylus, in 0.01 mm units) = 5–6: 4–5: 20–22: 2: 9–13. **Thorax** brownish black, grey microtrichose; mesoscutum microtrichose in dorsal view. Chaetotaxy: All setae and setulae black. Antepronotum with row of setae; proepisternum with about 6 setae; propleura with 2 setae; prosternum bare; acrostichals biserial and short (about 0.08 mm long, 8 setae in one row); dorsocentrals slightly longer, irregularly biserial and slightly diverging, ending in 1–2 longer prescutellar pairs; 1 long postpronotal and 3–4 small additional setae; 1–2 presutural supra-alar, 1 presutural intra-alar, no additional setae in presutural area; notopleuron with 3 long setae and 2–3 additional setae anteriorly; 1–2 postsutural supra-alar; 2–3 short pre-alars; 1 post-alar; 1 pair of scutellars; laterotergite with black setae. **Legs** including coxae brownish black, microtrichose, entirely black setose. *Fore leg*: Femur with short dorsal and ventral setae except several longer preapical posteroventral; tibia with short and soft ventral ciliation, posterodorsally with setae shorter than tibia depth and with 2–4 strong anterodorsals longer than tibia depth; first tarsomere as broad as tibia basally and narrower apically, both dorsally and ventrally with setae about as long as tarsomere depth, preapical setae conspicuous but rather short, similar setosity on tarsomeres 2–4; ratio of tibia: first tarsomere = 1.9–2.0. *Mid leg*: Femur dorsally short setose, anteriorly about base with somewhat longer setae, anteroventral row of densely set setae about as long as femur depth, longer on basal part, posteroventral sparse row of similarly long setae; tibia with a single very long anterodorsal seta in proximal third three times tibia depth and additional smaller seta), preapical seta equally long, 2–4 posterodorsal setae much shorter, two rows of ventral setae, at most, twice as long as tibia depth; first tarsomere narrow, both dorsally and ventrally short setose, preapical seta equally long as preapical tibial seta, remaining tarsomeres without long preapical setae. *Hind leg*: Femur with nearly complete row of anterodorsal setae almost as long as tibia depth, anteroventral row of setae equally long, posteroventrals present on proximal third of femur; tibia slightly swollen and flattened, with a row of rather strong ventral setae about as long as tibia depth on proximal part and shorter more distally, dorsally with two rows of setae slightly longer than tibia depth; first tarsomere slightly narrower than tibia, dorsally with several setae slightly shorter than tarsomere depth, ventrally and anteriorly with several short spines. Comb at tip of hind tibia without longer seta. **Wing** membrane almost clear, stigma brown, all veins brown, axillary angle acute; costal seta short; anal vein (CuA_1_) indistinct from about half of its length to nearly tip, tip again distinct. Halter brown, calypter grey to brownish grey, with black fringes. **Abdomen** brown, brownish grey microtrichose, tergites laterally very slightly sublustrous, all setae brown to black. Lateral setae on tergites 2–3 slightly shorter than corresponding segments, on following segments much shorter, marginals slightly longer. Sternites with pair of submedian posterior marginal setae, sternite 1 without setae. Genitalia (Figure 2A–E): Cercus in dorsal view with one long spine and additional 4–5 shorter spines subapically, basally with about 10 peg-like setae; epandrium rather short, about as long as high, with rounded tip covered with short setae; hypandrium long, its tip merging inside broadened phallus; phallus broadened in basal two thirds and very narrow apically. **Length:** Body 3.8–3.9 mm, wing 3.2–3.8 mm.

**Female:** Unknown.

**Remarks:** *Empis* (*Empis*) *manteigasensis* sp. nov. is a member of the *E*. (*E*.) *chioptera* group, *E. aestiva* complex. The most allied species is *E. aestiva* Loew, 1867, but mid tibia and first tarsomere with much longer preapical setae (scarcely longer than these tarsomeres depths in *E. aestiva*) and quite different genitalia as described in the key below (compare Fig. 2 with figures 214–217 in [2]).

**

***Empis* (*Empis*) *miroslavi* sp. nov.**

Zoobank link: urn:lsid:zoobank.org:act:A549791E-B24A-4AFF-AA2A-CBF792034A6F

(Figure 3A–D)

**Type material: HOLOTYPE** ♂, **Portugal**, 7 km E of Manteigas, nr. river, 580 m, sweeping, 40°24′42″ N, 7°28′4″ W, Barták M., 23.v.2008 (CULSP). **PARATYPES**: 1♂, same data as holotype—(CULSP).

**Diagnosis:** Small species of the *E*. (*Empis*) *chioptera* group (*E. aestiva* complex) with black body and black setae. Mid tibia with long preapical anterodorsal seta. Anal vein incomplete, indistinct in apical part. Epandrium elongate, with a fan of very long setae around tip. Phallus without basal thickening. Female unknown.

**Etymology:** The species is named after my son, Miroslav, who helped me collect the samples in Portugal.

**Figure 3:**
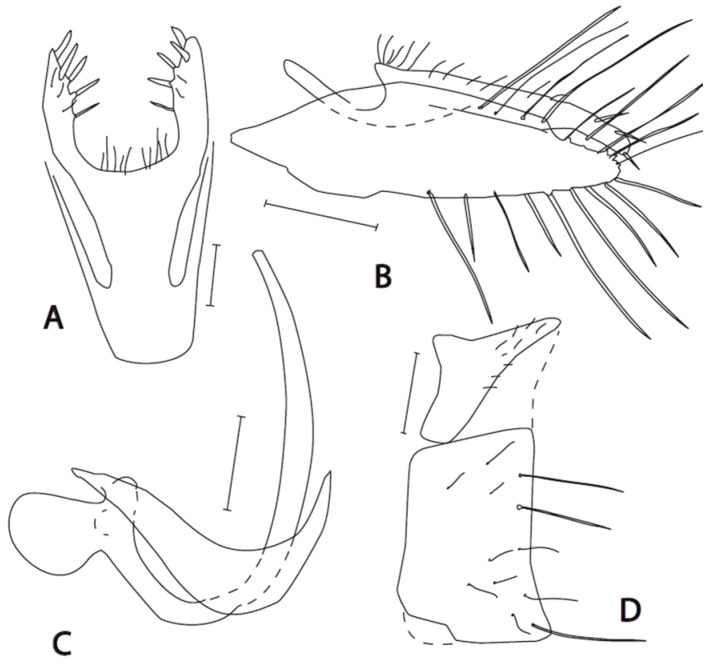
*Empis miroslavi* **sp. nov.** Postabdomen. **A** = Cerci, dorsal view; **B** = Epandrium + cercus; **C** = Phallus with hypandrium; **D** = Eighth abdominal segment. Paratype. Scale = 0.10 mm.

**Description. Male: Head** black, dark brownish grey microtrichose, holoptic, eyes meeting over long distance. Frons (small triangles just above antennae and below front ocellus) without setae. Dorsal half of eye with larger facets than ventral half. Ocellar setae black, half as long as frons. Occiput in dorsal third with irregularly arranged setae up to as long as ocellars, postocular row irregular in lower part. Face about 0.16 mm broad ventrally, microtrichose with lustrous ventral margin, without setae. Clypeus microtrichose except anterior corner, gena very narrow, sublustrous. Palpus black, with 3 setae in basal half and 2 rather long setae subapically. Labrum black, lustrous, 1.5–1.7 times longer than head height. Antenna black, both basal segments short setose; length of antennal segments (scape: pedicel: postpedicel: basal joint of stylus: last joint of stylus, in 0.01 mm units) = 6: 6: 18–20: 2: 8–9. **Thorax** brownish black, grey microtrichose; mesoscutum microtrichose in dorsal view. Chaetotaxy: All setae and setulae black. Antepronotum with row of setae; proepisternum with several setae; propleura with 2–3 setae; prosternum bare; acrostichals biserial and long (about 0.15 mm, 8–9 setae in one row); dorsocentrals slightly longer, irregularly biserial and slightly diverging, ending in 1–2 longer pairs, prescutellar pair very long; 1 long postpronotal and 4 smaller setae; 1 presutural supra-alar, 1 presutural intra-alar, 0–1 additional setae in presutural area; notopleuron with 3 long setae and 2–3 additional setae anteriorly; 1–2 postsutural supra-alar; 2–3 short pre-alars; 1 post-alar; 1 pair of scutellars; laterotergite with black setae. **Legs** including coxae brownish black, microtrichose, entirely black setose. *Fore leg*: Femur with short dorsal setae, complete posteroventral row of setae shorter than tibia depth; tibia with short and rather thick ventral ciliation including several longer preapicals, posterior ciliation soft and short (similar on first tarsomere), antero- and posterodorsally with heterogeneous setae up to 1.5 times longer than tibia depth; first tarsomere very narrow, dorsally with setae slightly longer than its depth, ventrally with spine like setae slightly longer, preapical setae conspicuous but rather short, about half as long as third tarsomere, similar setosity on following 2 tarsomeres; ratio of tibia: first tarsomere length = 2.2–2.4. *Mid leg*: Femur dorsally short setose, anterodorsal setae shorter than femur depth, 2 ventral rows of sparse setae (about 10 setae in anteroventral row) up to twice as long as femur depth; tibia with two anterodorsal setae in basal third, longest being 3 times longer than tibia depth, and similarly long preapical, 4 posterodorsal setae slightly longer than tibia depth, two rows of ventral setae somewhat longer than tibia depth; first tarsomere narrow, short setose, preapical setae short. *Hind leg*: Femur with nearly complete row of anterodorsal setae shorter than tibia depth, anteroventral row of setae with the longest one third before tip nearly twice as long as femur depth, more proximal and more distal setae gradually shorter; tibia slightly swollen and flattened, with ventral setae about as long as tibia depth, dorsally with several setae up to twice as long as tibia depth; first tarsomere slightly widened (about as broad as tip of tibia), dorsally with several setae slightly shorter than tarsomere depth, ventrally with several short spines. Comb at tip of hind tibia without longer seta. **Wing** membrane almost clear, stigma light brownish, all veins yellowish brown, axillary angle right; costal seta short; anal vein (CuA_1_) distinct (but depigmented) only in basal half. Halter brownish black, calypter grey to brownish grey, with black fringes. **Abdomen** brownish black, brownish grey microtrichose, tergites 2–5 laterally slightly sublustrous, all setae black. Lateral setae on tergites 2–3 slightly shorter than corresponding segments, on the following segments much shorter, marginals poorly differentiated. Sternites short setose except second segment, sternite 1 without setae but 1 pair of long setae on the base of sternite 2 (possibly sternite 1?). Genitalia (Figure 3A–D): Cercus peculiarly fused with subepandrial sclerite, in apical part with 4–5 subequally long spines; epandrium narrow and elongate, with very long setae on apical part both dorsally and ventrally; hypandrium simple bow shaped; phallus equally narrowing towards tip, without apparent broadenings. **Length:** Body 2.6–2.8 mm, wing 2.5–2.6 mm.

**Female:** Unknown.

**Remarks:** *Empis* (*Empis*) *miroslavi* sp. nov. is a member of the *E*. (*E*.) *chioptera* group, *E. aestiva* complex. It may be easily identified according to the key due to the combination of sparse anteroventral setae on the mid femur, long preapical anterodorsal seta on the mid tibia, and a peculiar fan of long setae around the tip of the epandrium. The female remains unknown; however, we found a female collected in the same sample with males of *E. manteigasensis* sp. nov. and *E. miroslavi* sp. nov., but its small size indicates the latter species. It has a completely black setose body, an incomplete anal vein, and peculiarly pennate hind femur ventrally (about 13 flattened setae distributed in apical 2/3 of femur with free spaces between them as wide as setae). The remaining parts of the legs are without flattened setae.

**

***Empis* (*Empis*) *moceki* sp. nov.**

Zoobank link: urn:lsid:zoobank.org:act:4E87589A-6A53-430F-AB6C-D38EBA977307

(Figure 4A–D)

**Type material: HOLOTYPE** ♂, **Bulgaria** centr., Izvor, 25 km SW Sofia, deciduous forest, B. Mocek, 12.vi.1994—(CULSP).

**Diagnosis:** Small species of the *E*. (*Empis*) *chioptera* group with black body and entirely black setae. Anal vein complete. Mid tibia with 8–9 long anterodorsal setae. Abdominal tergites 4–6 very short setose. Genitalia with relatively narrow cercus and rounded epandrium armed with very long setae.

**Etymology:** The species name is a patronym to honour the late Dr. Bohuslav Mocek, collector of the holotype.

**Figure 4:**
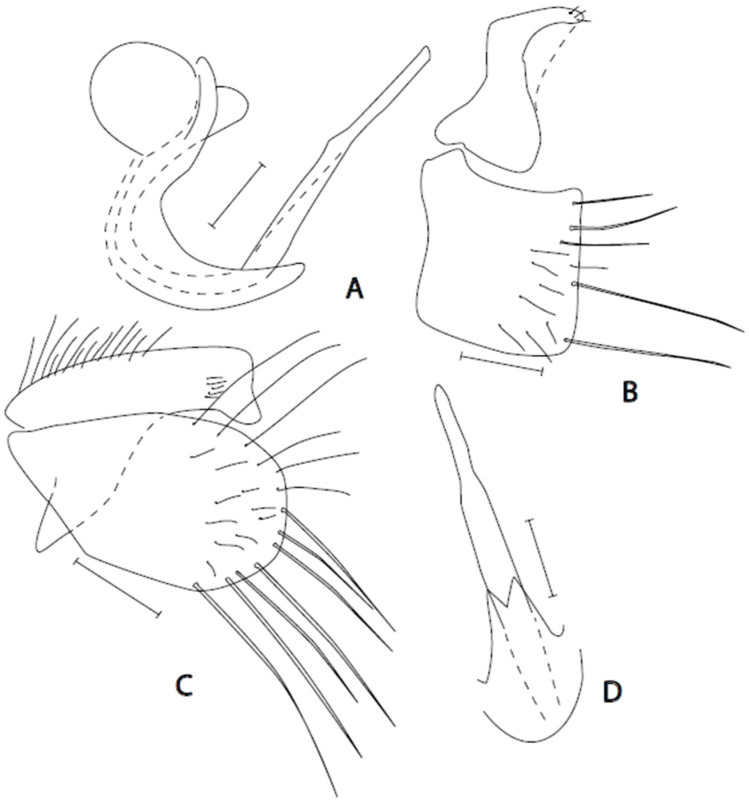
*Empis moceki* **sp. nov.** Postabdomen. **A** = Phallus with hypandrium; **B** = Eighth abdominal segment; **C** = Epandrium + cercus; **D** = Hypandrium + phallus, ventral view. Holotype. Scale lines = 0.10 mm.

**Description. Male: Head** black, grey microtrichose, holoptic, eyes meeting over long distance. Frons (small triangles just above antennae and below front ocellus) without setae. Dorsal half of eye with distinctly larger facets than ventral half. Ocellar setae broken in the only specimen at hand. Occiput rather densely covered with long fine setae, longer than postpedicel length. Face about 0.18 mm broad ventrally (slightly collapsed), microtrichose with lustrous ventral margin, without setae. Clypeus lustrous, medially microtrichose, gena very narrow. Palpus black, with several short setae apically. Labrum black, lustrous, 1.3–1.4 times longer than head height. Antenna black, both basal segments short setose; length of antennal segments (scape: pedicel: postpedicel: basal joint of stylus: last joint of stylus, in 0.01 mm units) = 8: 7: 17: 2: 14. **Thorax** black, grey microtrichose; mesoscutum rather light grey in anterior view and darker in posterior view. Chaetotaxy: All setae and setulae black. Antepronotum with row of setae; proepisternum with 4 setae; prosternum bare; propleura with 3 setae; acrostichals biserial, slightly divergent and medium long (about 0.13 mm, slightly longer than distance between rows of setae); dorsocentrals equally long, irregularly arranged, more than biserial anteriorly, not separated from other fine setae laterad of them, ending in 3 long and strong prescutellar pairs; 1 long postpronotal and about 10 smaller setae; 1 strong presutural supra-alar surrounded by more than 10 fine small setae, 4 setae in praesutural intra-alar area; notopleuron with 3 long and strong setae and 10 additional setae anteriorly; 2 pre-alars; 2 postsutural supra-alars (posterior one strong and long); 1 post-alar; 2 pairs of scutellars (inner pair long and crossed, outer pair smaller and finer); laterotergite with long and dense black setae. **Legs** including coxae black, coxae microtrichose, other parts of legs sublustrous to lustrous, entirely black setose. *Fore leg*: Femur dorsally short setose, anteroventrally almost bare, posteroventral setae short except several preapicals; tibia with very short ventral ciliation, posterodorsally with setae up to twice as long as tibia depth; tarsus including first tarsomere narrow, short setose, preapicals unremarkable; ratio of tibia: first tarsomere length = 2.0. *Mid leg*: Femur dorsally short setose, anteroventrally with row of about 17 strong setae more than twice as long as femur depth, gradually shorter apically, posteroventrally with setae slightly shorter and finer; tibia with 8–9 anterodorsal setae (on basal two thirds of tibia) up to three times tibia depth, and one subequally long preapical, posterodorsal setae much shorter, two ventral rows of setae twice as long as tibia depth; first tarsomere with rather long anterodorsal preapical seta 2/3 as long as following tarsomere and strong ventral spines, subbasal one twice as long as tarsomere depth. *Hind leg*: Femur with 2–3 extremely long and strong anterodorsal setae in basal half more than 3 times longer than femur depth, posteriorly in apical half with densely set protruding short setae, other setae short; tibia slightly widened and flattened, with ventral setae slightly shorter than tibia depth, dorsal setae in apical part slightly longer than tibia depth, shorter in basal part; first tarsomere narrow, dorsally with 5–6 setae (including preapicals) up to twice as long as tarsomere depth, ventrally with spines longer than tarsomere depth; remaining tarsomeres unremarkable. Comb at tip of hind tibia without longer seta. **Wing** membrane clear, anal vein (CuA_1_) complete, axillary angle sharply acute; costal seta absent. Halter black, calypter dark brownish black with black fringes. **Abdomen** blackish brown, grey microtrichose dorsally, tergites lustrous laterally, sternites microtrichose except contrastingly lustrous last sternite, all setae black. Lateral setae on tergites 1–3 medium long, on tergites 4–6 very short (in dorsal view, about 0.05 mm long and 5 times shorter than length of their segments), dorsal setae very short, posterior marginal setae not differentiated, tergite 8 modified and very short setose. Most sternites with a pair of long setae, sternite 8 long setose, sternite 1 without setae. Genitalia (Figure 4A–D): Cercus narrow, apically slightly broadened, on basal half dorsally rather long setose; epandrium ovate, with very long setae in apical part both dorsally and ventrally; phallus almost equally narrow and straight, in ventral view slightly broadened on basal half; hypandrium short, apically shallowly V-shaped fork. **Length:** Body 3.7 mm, wing 3.3 mm.

**Female:** Unknown.

**Remarks:** *Empis* (*Empis*) *moceki* sp. nov. is a member of the *E*. (*E*.) *chioptera* group but genitalia are different, belonging to neither *chioptera* (cercus narrow and elongated) nor *aestiva* (cercus without basal humb and without internal or apical spines) complexes. Moreover, distinct features, besides those given in the key, are the following: densely setose presutural mesoscutum laterad from dorsocentrals, long and densely long setose mid tibia, 2–3 very long anterodorsal setae on hind femur, and very short setose tergites.

**

***Empis* (*Empis*) *serviae* sp. nov.**

Zoobank link: urn:lsid:zoobank.org:act:48FC915A-13D3-4924-84F4-8C7A41E1B01D

(Figure 5A–E)

**Type material: HOLOTYPE** ♂, **Spain**, Fragas do Eume NP, MT2 (along brook), 43.413, −8.064, 60 m, Garcia, Ševčík, 30.v.–20.vi.2019 (CULSP). **PARATYPES**: 1♂, same data as holotype; 1♂, same locality but 16–30.v.—(CULSP); 2 males, **Spain**, Santander, Aliva-Magrovejo, 11.vi.1981, meadow, leg. Ragnar Hall; 2 males, Spain, Leon. Oseja de Sajambre, open meadow in beach oak forest, 22.vi.1988, leg. P. Hall (HNRS)—NMP.

**Diagnosis:** Small species of *E*. (*Empis*) *chioptera* group (*aestiva* complex) with black body and entirely black setae. First fore tarsomere narrow with short preapical setae, mid tibia with short preapical anterodorsal seta. Anal vein incomplete.

**Etymology:** The species is named after the collector of this type series, María José Servia García, who operated Malaise traps in Fragas do Eume National Park.

**Figure 5:**
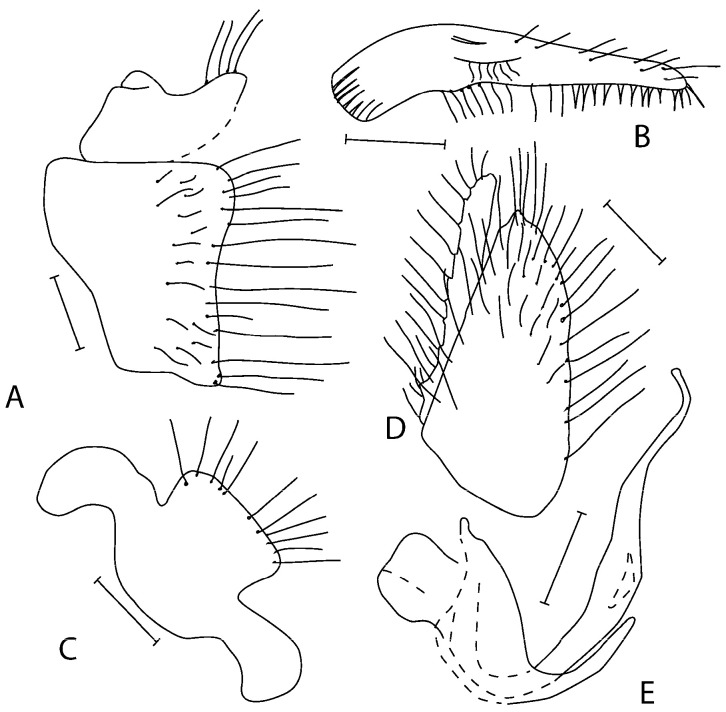
*Empis serviae* **sp. nov.** Postabdomen. **A** = Eighth abdominal segment; **B** = Cercus, dorsal view; **C** = Tergite 8, anterodorsal view; **D** = Epandrium + cercus; **E** = Phallus with hypandrium. Paratype. Scale lines = 0.10 mm.

**Description. Male: Head** black, dark brownish grey microtrichose, holoptic, eyes meeting over long distance. Frons (small triangles just above antennae and below front ocellus) without setae. Dorsal half of eye with distinctly larger facets than ventral half. Ocellar setae black, slightly less than half as long as frons. Occiput in dorsal third with irregularly arranged setae, postocular row nearly complete but irregular in lower part; dorsal postocular setae slightly shorter than ocellars. Face about 0.12 mm broad ventrally, microtrichose with lustrous ventral margin, without setae. Clypeus lustrous, gena very narrow, sublustrous. Palpus black, with several rather long setae basally and apically. Labrum black, lustrous, 1.4–1.6 times longer than head height. Antenna black, both basal segments short setose; length of antennal segments (scape: pedicel: postpedicel: basal joint of stylus: last joint of stylus, in 0.01 mm units) = 5: 6: 20–24: 1–2: 11–14. **Thorax** black, dark brownish grey microtrichose; mesoscutum sublustrous in dorsal view. Chaetotaxy: All setae and setulae black. Antepronotum with row of setae; proepisternum with 7–9 setae; prosternum bare; propleura setose; acrostichals biserial and long (about 0.17 mm, 7–9 setae in one row); dorsocentrals equally long, irregularly biserial ending in 2 longer prescutellar pairs; postpronotal not much differentiated from several additional setae; 1–2 presutural supra-alar, 1 presutural intra-alar and additional 2–3 setae nearby; notopleuron with 3 long and strong setae and with several additional rather long setae anteriorly; 1 postsutural supra-alar; 2–3 rather long prelars; 1 post-alar; 1 pair of scutellars; laterotergite with black setae. **Legs** including coxae brownish black, microtrichose, entirely black setose. *Fore leg*: Femur with short dorsal and ventral setae except several preapical; tibia with very short ventral ciliation, posterodorsally with setae up to twice tibia depth; first tarsomere about as broad as tibia, dorsally with setae about as long as tarsomere depth, ventrally with fine and short setae, apically with rather short setae (about one third as long as tarsomere), similar short apical setae also on following three tarsomeres; ratio of tibia: first tarsomere length = 2.1–2.3. *Mid leg*: Femur dorsally short setose, anteroventrally with irregular rather dense row of setae slightly shorter than femur depth on basal half and nearly twice longer on apical half, posteroventrally with setae slightly longer in basal half than more apically; tibia with 2–3 anterodorsal setae (on basal half of tibia) up to three times tibia depth, posterodorsal setae shorter, more numerous and more equally distributed, two ventral rows of setae twice as long as tibia depth, preapicals rather short (about 1.5 times tibia depth); first tarsomere dorsally short setose, ventrally with several spines as long as tarsomere depth, preapical setae short. *Hind leg*: Femur with nearly complete row of anteroventral setae up to a long as femur depth and several anterodorsals, posteroventral setae short, in apical third rather spinose and dense, forming a comb of about 10 setae; tibia slightly flattened, with ventral setae slightly shorter than tibia depth, dorsal setosity rather long, setae up to twice as long as tibia depth; first tarsomere slightly swollen but narrower than tip of tibia, dorsally with several longer setae similar to those on tibia. Comb at tip of hind tibia without longer seta. **Wing** membrane clear to slightly greyish, veins brown, axillary angle right; costal seta short; anal vein (CuA_1_) indistinct in apical third except tip. Halter black to dark brown, calypter dark brown with black fringes. **Abdomen** blackish brown, grey microtrichose, tergites in certain aspect slightly sublustrous, all setae black. Lateral posteromarginal setae on tergites longer than segments, discal slightly shorter, tergite 8 modified (Figure 5A,C). Most sternites with a pair of very long posteromarginal setae much longer than sternites length, sternite 8 with long and dense setosity, sternite 1 without setae. Genitalia (Figure 5B,D,E): Cercus elongate and narrow, base of dorsal margin slightly protruding, covered with several spine-like setae, posterior half covered with about 30 interior equally long spines arranged in several rows; epandrium elongate oval, densely covered with medium long setae. **Length:** body 2.8–3.1 mm, wing 3.1 mm.

**Female:** Unknown.

**Remarks:** *Empis* (*Empis*) *serviae* sp. nov. is a member of the *E*. (*E*.) *chioptera* group, *aestiva* complex. The most allied species are *E. praevia* Collin, 1927, and *E. levis* Loew, 1873; differences are apparent from the key.

**

***Empis* (*Empis*) *troyanensis* sp. nov.**

Zoobank link: urn:lsid:zoobank.org:act:A9366F7C-4E79-4CD3-A973-B3A919073856

(Figure 6A–D)

**Type material: HOLOTYPE** ♂, **Bulgaria**, Troyan pass, meadow, sw [= sweeping vegetation], 42.778, 24.611, 1550 m, Barták, Kubík, 22.vi.2017 (CULSP). **PARATYPES**: **Bulgaria**: 2♂, same data as holotype; 4♂, Troyan pass, nr. brook, sw, 1400 m, 42.774, 24.618, Barták, Kubík, 22–24.vi.2017; 1♂, 13 km SW of Troyan, wood + meadow, sw, 1350 m, Barták, Kubík, 15–22.vi.2017; 6♂, 5♀, 5 km W of Smolyan, clearing in wood, 1260 m, 41.569, 24.632, Barták, Kubík, 23.vi.2018; 1♂, same locality, MT [=Malaise trap], 14–26.vi.2019; 1♂, 1♀, Pamporovo, 1300–1600 m, sw + pt [=sweeping + pan traps], 41.639, 24.697 ± 2 km, Barták, Kubík, 14–18.vi.2018; 1♂, Shipka pass, edge of Fagetum, sw, 1240 m, 42.748, 25.335, Barták, Kubík, 21.vi.2017; 1♂, 6 km E of Dospat, pasture + flowers, 41.663, 24.222, Barták, Kubík, 22–23.vi.2019; 3♂, 2♀, Rhodopes, 25 km SSW of Plovdiv, 1590 m, meadow, 41.935, 24.679, Barták, Kubík, 20.vi.2016; 5♂, 3–9 km SW of Bansko, 1100–1800 m, Sw + Pt, 41.789, 23.45 ± 3 km Barták, Kubík, 21–22.vi.2018; **Slovakia**: 1♂, Muránska planina, Rosiarka, Malaise trap, peat-bog, 48.713, 19.825, 920 m, Ševčík, Roháček, 26.v.-21.vi.2022; 1♂, 1♀, Zadná Polana, spruce wood, Malaise trap, 1250 m, 48°39′40″ N, 19°29′50″ E, Ševčík, Roháček, 6.v.-3.vii.2006; **Italy**: 1♂, Passo nigra, meadow + wood, 1700 m, 46°26′39″ N, 11°35′18″ E, Barták, 5.vii.2011; 2♂, 1♀, Weisslahnbad, edge of forest, 1400 m, 46°28′40″ N, 11°34′11″ E, Barták, 4.vii.2011—(CULSP).

**Diagnosis:** Small species of *E*. (*Empis*) *chioptera* group (*chioptera* complex) with black body and entirely black setae, 4 scutellars. Fore femur with very long posteroventral setae, in apical third about twice as long as femur depth. Fore tarsomeres with long apical setae. Abdominal sternites short setose. Hypandrium without setae.

**Etymology:** The species epithet, *troyanensis*, is derived from Troyan in the Central Balkans, one of the type localities.

**Figure 6:**
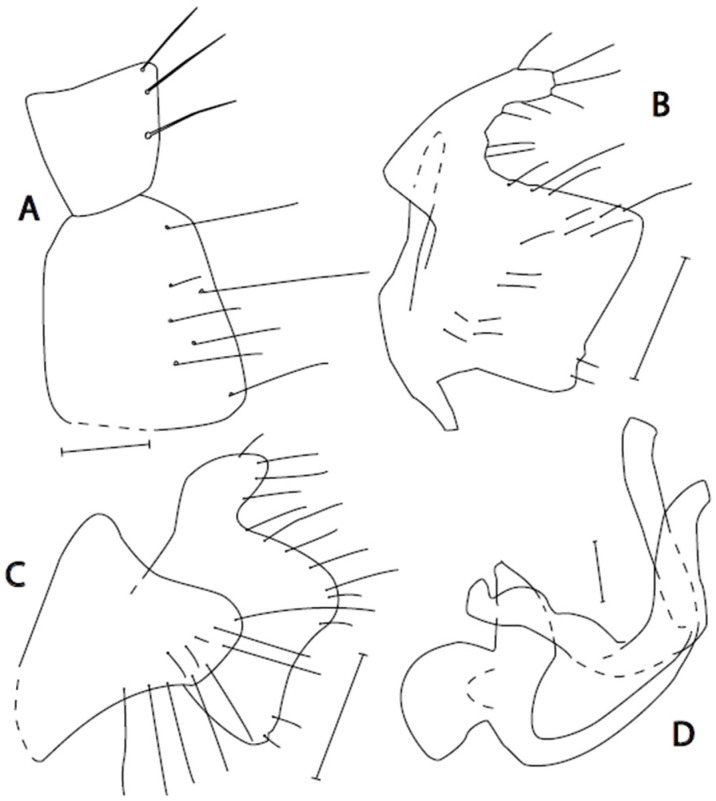
*Empis troyanensis* **sp. nov.** Postabdomen. **A** = Eighth abdominal segment; **B** = Cercus; **C** = Epandrium + cercus; **D** = Phallus with hypandrium. Paratypes. Scale lines = 0.10 mm.

**Description. Male: Head** black, dark grey microtrichose, holoptic, eyes meeting over long distance. Frons (small triangles just above antennae and below front ocellus) without setae. Dorsal half of eye with distinctly larger facets than ventral half. Ocellar setae black, less than half as long as frons. Occiput in dorsal third with two rows of long setae, in middle part more densely and shorter setose, ventral part with irregularly arranged, long and fine black setae. Face about 0.16 mm broad ventrally, microtrichose with lustrous narrow ventral margin, without setae. Clypeus with two lustrous stripes, medially microtrichose, gena very narrow, microtrichose. Palpus black, with 2–4 rather long setae basally and another 1–3 setae apically. Labrum black, lustrous, 1.5–1.6 times longer than head height. Antenna black, both basal segments short setose; length of antennal segments (scape: pedicel: postpedicel: basal joint of stylus: last joint of stylus, in 0.01 mm units) = 4: 5–6: 22–24: 2–3: 12. **Thorax** black, dark grey microtrichose; mesoscutum sublustrous in dorsal view. Chaetotaxy: All setae and setulae black. Antepronotum with row of setae; proepisternum with 8–10 setae; prosternum bare; propleura setose; acrostichals biserial and long (about 0.15 mm, 8–10 setae in one row); dorsocentrals equally long, irregularly biserial ending in 2–3 longer prescutellar pairs; 1 long postpronotal and several smaller setulae; presutural supra-alar, presutural intra-alar and pre-alar not differentiated from 2 to 3 setae; notopleuron with 3 long and strong setae and with several additional setae anteriorly; 1–2 postsutural supra-alars; 1 post-alar; 2 pairs of long scutellars (outer pair smaller); laterotergite with black setae. **Legs** including coxae brownish black, microtrichose, entirely black setose. *Fore leg*: Femur with short dorsal and anteroventral setae except several preapical, posteroventral setae very long, on apical half up to twice as long as femur depth; tibia with very short ventral ciliation, posterodorsally with setae up to twice as long as tibia depth, some of them hooked; first tarsomere slightly widened, dorsally with setae twice as long as tarsomere depth, ventrally with strong setae as long as tarsomere depth, apically with long setae, similar long apical setae also on following three tarsomeres; ratio of tibia: first tarsomere length = 1.4–1.5. *Mid leg*: Femur dorsally short setose, anteroventrally with irregular sparse row of setae up to twice as long as femur depth, posteroventrally with setae more than twice as long as femur depth; tibia with 3–4 anterodorsal setae (on basal two thirds of tibia) up to three times longer than tibia depth, and one subequally long preapical, posterodorsal setae much shorter, two ventral rows of setae twice as long as tibia depth; first tarsomere dorsally short setose, ventrally with rather long spines, preapical setae long (anterodorsal nearly half as long as tarsomere length). *Hind leg*: Femur with 2–3 anterodorsal and 3–4 ventral setae longer than femur depth, posteroventral setae short and sparse; tibia with ventral setae slightly shorter than tibia depth, dorsal setosity variable, some specimens almost without dorsal setae except preapicals, some with clearly differentiated 1–3 setae longer than tibia depth; first tarsomere swollen, nearly twice broader than tip of tibia (and distinctly broader than first fore tarsomere), dorsally with several longer setae over whole length; remaining tarsomeres unremarkable. Comb at tip of hind tibia without longer seta. **Wing** membrane clear to slightly milky white, vein C (to R_5_) and R veins yellowish brown, remaining veins white to light brownish yellow, anal vein (CuA_1_) complete, axillary angle slightly acute; costal seta very short or absent. Halter black to dark brown, calypter grey with black fringes. **Abdomen** blackish brown, grey microtrichose, tergites 3–5 laterally sublustrous, all setae black. Lateral setae on tergites shorter than segments (except somewhat longer on tergites 1–2), dorsal setae very short, posterior marginal setae not differentiated. Sternites very short setose, sternite 1 without setae. Genitalia (Figure 6A–D): Hypandrium long (almost as long as apical straight part of phallus), bare and desclerotized medially, evenly narrowed towards tip; epandrium short, subtriangular with rounded tip, setae around apex almost half as long as epandrium; cercus larger than epandrium, of usual *chioptera* type, lower lobe almost square; phallus rather short and thick, narrowed at medial bend, slightly bowed at apex (without preapical S-shaped bend). **Length:** Body 3.3–4.2 mm, wing 3.6–4.7 mm.

**Female:** Dichoptic, all facets equal in size, frons about 0.15 mm broad with rows of several rather long black setae on each side. Labrum nearly twice as long as head height. Mesonotum similarly setose as in male, only setae much shorter (acrostichals about 0.08 mm, dorsocentrals slightly longer). *Fore leg*: Femur very short setose; tibia short setose, posterodorsally with homogeneous setae slightly shorter than tibia depth, no pennation first tarsomere short setose dorsally and somewhat longer setose ventrally, tibia 1.7–1.8 x longer than first tarsomere. *Mid leg*: Femur short setose dorsally, ventrally with two rows of setae at most (in anteroventral row before tip) as long as femur depth; tibia with several setae at most as long as tibia depth; first tarsomere narrow and ventrally with stronger setae slightly longer than tarsomere depth. *Hind leg*: Femur slightly flattened, ventrally very short setose on basal half, apically with setae slightly longer than femur depth, sometimes with tendency to be slightly flattened, dorsally rather densely fringed with simple setae shorter than femur depth; tibia with ciliation dorsally and ventrally shorter than tibia depth, dorsally with 0–4 setae slightly longer than tibia depth and two long preapical setae; first tarsomere narrow, short setose, dorsally with 0–3 setae slightly longer than tarsomere depth. Abdomen with black and very short setae (posteromarginals not differentiated), sternites 1–5 and tergites 2–5 very slightly sublustrous. **Length:** Body 3.1–3.9 mm, wing 3.4–4.2 mm.

**Remarks:** *Empis* (*Empis*) *troyanensis* sp. nov. is a member of the *E*. (*E*.) *chioptera* group, very close to *E*. (*E*.) *kamyshanovensis* Kustov & Shamshev, 2013. However, besides the characteristics given in the key, the newly described species has a longer labrum and postpedicel. The female is similar to *Empis* (*Empis*) *mariae* Syrovátka, 1991; differences are given in the key.

**

**Notes on other species of *chioptera* group**

**

Only those females collected with the males are recorded due to identification uncertainty.

**

***Empis* (*Empis*) *mariae* Syrovátka, 1991**

Material examined: 2 males, **Switzerland**, ZH, 500 m, Sihlwald, 22.viii.1998, leg. B. Merz; 1 male, **Romania**, Trans. Alps, Mnt. Bucegi, Busteni, 1200 m, 16.vii.1970, M. Chvála; 3 males, 1 female, **Austria**, Sulzgau, along river, 47.33N/ 13.11E, 30.vii.1988, M. Barták; Austria, Styria, Admont, meadows, 640 m, 4.viii.2010, leg. M. Chvála; 3 males, 1 female, Styria, Gesäuse, Admont, Kematengraben, 1000 m, 22.ix.2006, leg. M. Chvála—NMP and CULSP.

Additions and corrections to original description [21]. **Male**: Length of body 2.6–3.0 mm, wing 2.9–3.3 mm (2.4–3.0 mm/ 3.0–3.3 mm in [21]). All body setae black (some of them erroneously described by [21] as “fair” or with “fair tips”). Head holoptic, eyes meet over long distance, dorsal facets distinctly larger than ventral ones. Ocellar setae slightly shorter than dorsal postocular setae (smaller than half of length of frons). Occiput almost bare dorsally behind postocular row, below neck with longer and more densely arranged black setae. Face entirely microtrichose, clypeus lustrous. Labrum 1.3–1.5 times longer than head height. Gena narrow and lustrous. Palpus with 3–4 setae on basal part and 1–3 setae subapically. Mesoscutum rather light brownish grey microtrichose. Total of 1 postpronotal seta and several shorter setae; acrostichals sparse (about 10 setae in one row) and short (about 0,10 mm); dorsocentrals irregularly biserial, diverging, with only 1–2 setae outside rows in presutural area beside 1 intra- and 1 supra-alar; postsutural area with 2 supra-alars, 2–3 short pre-alars, 1 post-alar, 4 scutellars (outer pair small). *Fore leg*: Femur with short setae, antero- and postero- ventrals very short except several preapicals; tibia with posterodorsal setae up to twice as long as tibia depth and relatively fine posteroventrals about as long as tibia depth; first tarsomere slightly swollen, ratio of tibia: tarsomere = 1.3–1.7, preapicals on first three tarsomeres about as long as length of third tarsomere. *Mid leg*: Femur with antero- and postero- ventral setae almost twice as long as femur depth; tibia with 3 anterodorsal setae in basal two thirds and one preapical and 3–4 ventral setae, all three times longer than tibia depth and similar seta on first tarsomere. *Hind leg*: Femur with a row of fine posterior setae up to as long as femur depth; first tarsomere dorsally with 0–2 setae nearly as long as tarsomere depth (beside preapicals). Wing clear, almost milky white, costa dark from Sc to tip, R_1_, and R_4+5_ often darkened, remaining veins colourless or yellowish, pterostigma hyaline, squama yellowish brown, black setose; costal seta very short. Abdomen blackish brown, microtrichose in dorsal view and lustrous to sublustrous on sides of tergites and on sternites (except microtrichose last two sternites), black setose. Tergites 2–4 on sides with setae almost as long as their segments, posteromarginals not differentiated. Sternites very short setose, sternite 1 without setae. Genitalia properly illustrated by [21]: cercus large, lower lobe almost square-shaped with rounded dorsal angle, ventral angle with ventrally protruding apex; epandrium sharply triangular, with a single very long apical seta and several smaller setae ventrally; hypandrium relatively thick, without markedly protruding dorsal bent part.

**Female**: Head dichoptic. All facets of equal size. Frons about 0.15 mm wide, with several rather long setae on each side. Postocular setae including ocellars rather thick. Mesoscutum sparsely setose as in male, setae shorter. *Fore leg*: femur very short setose; tibia posterodorsally with setae shorter than tibia depth, similar setosity on first tarsomere. *Mid leg*: Femur dorsally with dense dorsal fringe of short simple setae shorter than femur depth, ventral setae similarly short but sparser; tibia short setose, with several anterodorsal and posterodorsal setae as long as tibia depth. *Hind leg*: Femur slightly flattened, with ciliation similar to mid femur; tibia flattened, similarly setose as hind femur. First tarsomeres of all legs unremarkable. Wing light brown, pterostigma not apparent. Abdomen brownish black, sublustrous (including tergites in dorsal view and sternites), last three segments microtrichose, all setae black and very short. Length of body 2.7–3.3 mm, wing 3.2–3.6 mm.

**Differential diagnosis**. Males cannot be confused with any other known species due to the peculiar shape of the epandrium (see Fig. 2 in Syrovátka [21]). Females are rather similar to *E. troyanensis* sp. nov.; differences are given in the key.

**Distribution**: Czech Republic, South Germany, Switzerland, Austria, Romania. **Dates**: vii.–ix.

**

***Empis* (*Empis*) *morosa* Meigen, 1822**

(Figure 7A–C)

Material examined: total of 2 males, 1 female, **France**, Col de Tourniol, 1050 m, pasture + wood, SW, 44°55′06″ N, 5°11′04″ E, M. Barták, 26.v.2006; 1 male, **Czech Republic**, Vinařická horka, sweeping, 350–400 m, 50°11′03″ N, 14°04′59″ E, M. Barták, 14.iv.2007; 1 male, 1 female, Podyjí NP, Liščí skála, Qurcetum, 420 m, PT, 48°49′48″ N, 15°56′28″ E, M. Barták, Š. Kubík, 24–26.vi.2001; 1 male, Bílina, Holibka, hilltop steppe, YPWT, 50.31.20N, 13.49.40E, 420 m, M. Barták, 9–23.iv.1997—all CULSP; 1 male, **Switzerland**, VS, Leuk-Platten, 19.v.1996, Merz & Baechli leg. (*E. morosa* det. Chvála)—NMP.

**Figure 7:**
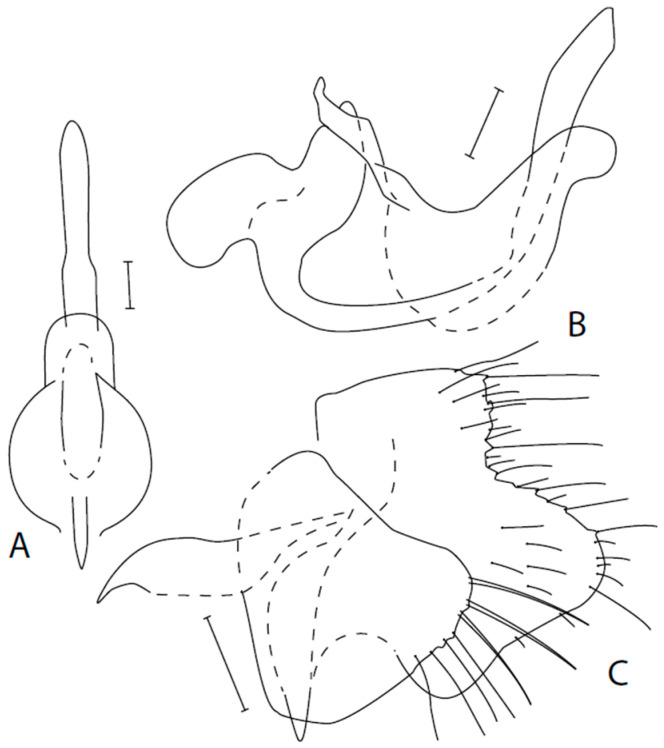
*Empis morosa* Meigen. Postabdomen. **A** = Hypandrium + phallus, ventral view; **B** = Hypandrium + phallus; **C** = Epandrium + cercus. Scale lines = 0.10 mm.

Additions and corrections to redescription of lectotype by Syrovátka & Chvála [22]. **Male**: Length of body 3.9–4.1 mm, wing 3.5–4.5 mm (3.6 mm/3.8 mm in [22]). Ocellar setae slightly smaller than dorsal postocular setae (smaller than half of length of frons). Labrum 1.5–1.8 times longer than head height (twice as long in [22]). Face entirely microtrichose, labrum lustrous, gena narrow and lustrous. Palpus with 3 setae on basal part and 1–3 setae subapically. Mesoscutum rather light brownish grey microtrichose, with somewhat darker stripes under rows of setae. 1–2 postpronotal setae; acrostichals numerous (about 15 setae in one row) and short (about 0,10 mm); dorsocentrals irregularly 2–3 serial, strongly diverging, with numerous setae outside rows of setae (about 20 setulae in presutural area between dorsocentrals and notopleuron); 4 scutellars, rarely with additional smaller seta(e). *Fore leg*: Femur with a row of posteroventral setae longer than femur depth; tibia with posterodorsal setae about twice as long as tibia depth; length ratio of tibia: first tarsomere = 1.3–1.7. *Mid leg*: Tibia with 3 anterodorsal setae in basal half (or basal two thirds) and one preapical and 3–4 ventral setae, all about twice as long as tibia depth and similar seta on first tarsomere. Wing clear, radial veins brown, remaining brownish yellow to yellowish brown; costal seta absent. Abdomen brownish black, microtrichose, black setose. Tergites 2–5 on sides with setae almost as long as their segments, posteromarginals not differentiated. Sternites very short setose. Genitalia (Figure 7A–C): Cercus very large, lower lobe almost square-shaped with rounded dorsal and ventral apical angles; epandrium relatively small, rounded apically, with several medium long setae on apex; hypandrium largely desclerotised medially, with downcurved tip in lateral view, in ventral view with sudden constriction in distal part.

**Female**: Head dichoptic. All facets of equal size. Frons about 0.20 mm wide, with about 10 rather long setae on each side. Mesoscutum rather densely setose as in male, only setae shorter. *Fore leg*: Femur short setose, posteroventral setae scarcely one third as long as femur depth (except several longer preapicals); tibia dorsally at least in apical half with pennate setae slightly shorter than tibia depth, ventrally short setose, near apex with several flattened setae; first tarsomere with dorsal short pennation similar to tibia. *Mid leg*: Femur dorsally with pennate ciliation slightly shorter than femur depth, posteroventrally with similar pennation except on basal third; tibia short setose, anterodorsally with slightly flattened setae. *Hind leg*: Femur with pennation similar to mid femur; tibia very slightly broadened, short setose. Mid and hind first tarsomeres unremarkable. Wing brown, pterostigma slightly darker, costal seta absent. Abdomen brown, very short setose, microtrichose except lustrous sides of segment 8. Length of body 3.5–3.9 mm, wing 3.5–4.1 mm.

**Differential diagnosis**. Males can be easily differentiated from other allied species by the peculiar shape of the hypandrium, as described above. Females can be distinguished according to the key. Moreover, both sexes have rich additional setation, especially in the hind part of the presutural area (between presutural supra-alar seta and dorsocentrals).

**Distribution**: Czech Republic, France, Switzerland. **Dates**: iv.–iv.

**

***Empis* (*Empis*) *scaura* Loew, 1867**

Material examined: total of 1 male, **Austria**, Styria, Admont, Saugraben meadows, 700 m, 23.vi.2007, leg. M. Chvála; 3 males, 1 female, Styria, Gesäuse, Keiserau, forest road, 1250 m, 2.vii.2006, leg. M. Chvála; 1 male, 1 female, same locality, 1000 m—NMP and CULSP.

Additions and corrections to redescription by Syrovátka [20]. **Male**: Length of body 3.5–4.1 mm, wing 3.9–4.3 mm (3.2–3.5 mm/3.8–3.9 mm in [20]). All body setae black (some of them erroneously described as “fair” by [20]). Head holoptic, eyes meet over long distance, dorsal facets distinctly larger than ventral ones. Ocellar setae half as long as frons (slightly longer than dorsal postocular setae). Occiput sparsely setose dorsally, setae almost in two regular rows), more densely and longer setose ventrally and below neck. Face entirely microtrichose, clypeus lustrous. Labrum 1.2–1.4 times longer than head height. Gena narrow and microtrichose. Palpus with 2–3 setae on basal part and 2–3 setae subapically. Mesoscutum rather light grey microtrichose in anterior view and dark black and sublustrous in dorsal view, scutellum and prescutellar depression rather light grey similarly as pleurae. Pronotum, propleura, and proepisternum with rather long setae, prosternum without setae. One postpronotal seta and 8–10 shorter setae; acrostichals sparse (about 10 setae in one row), biserial and long (about 0,15 mm); dorsocentrals irregularly biserial, diverging in middle of rows; only 2–3 setae outside rows in presutural area beside 1 intra- and 1 supra-alar (erroneously described as “posthumeral part of mesonotum with many dark hairs” by [20]); in postsutural area 1–2 supra-alars, 2–4 pre-alars, 1 post-alar; 4 scutellars (outer pair slightly shorter). *Fore leg*: Femur with complete row of posteroventral setae in apical third almost twice as long as femur depth; tibia with posterodorsal setae twice as long as tibia depth; first tarsomere swollen, dorsally with setae longer than those on tibia, ratio of tibia: first tarsomere = 1.5–1.6, preapicals not much longer than remaining dense and long setation; tarsomeres 2–3 distinctly swollen, with similarly long dorsal setation. *Mid leg*: Femur with antero- and posteroventral setae almost twice as long as femur depth, anteroventral row duplicated with 1–2 rows of additional setae inserted more anteriorly; tibia with anterodorsal setae three times longer than tibia depth; first tarsomere dorsally with fine setae and ventrally with coarse setae longer than tarsomere depth. *Hind leg*: Femur in apical third with 2–3 anterodorsal and 2–4 anteroventral setae longer than femur depth, in basal part much ciliation; first tarsomere swollen, (postero-)dorsally with setae slightly longer than tarsomere depth. **Wing** clear, costa dark from Sc to tip, R veins brownish, remaining veins brownish yellow to whitish yellow, pterostigma hyaline, squama grey to brown, black setose; costal seta very short. Abdomen brownish black, rather light grey microtrichose, black setose. Tergites 2–5 on sides with setae about as long as their segments, posteromarginals poorly differentiated. Sternites very short setose, sternite 1 without setae. Genitalia properly illustrated by [20] (Figs 12 D–G): cercus of typical *chioptera*-type; epandrium short and rounded, posteriorly with setae subequally long as epandrial lamella; hypandrium boat-shaped, with several rather long setae; phallus rather thick, with only very slight widening and curve.

**Female**: Head dichoptic. All facets of equal size. Frons about 0.20 mm wide, with several rather long setae on each side. Postocular setae including ocellars rather thick. Mesoscutum setose as in male, setae shorter. Legs as described by [20]. Wing distinctly greyish clouded in anteroapical half and almost clear in posterobasal part. Abdomen blackish brown, all setae black and very short. Length of body 3.4–3.9 mm, wing 3.9–4.4 mm (3.2–3.5/3.8–3.9 in [20]).

**Differential diagnosis**. Males cannot be confused with any other known species due to the setose hypandrium (see Fig. 12 E by [20]) and black abdominal setae, except for *E. dasyprocta* (with yellow abdominal setae). If these setae are overlooked in specimens with hypandrium hidden under the eighth segment, then it should be compared with *E. troyanensis* sp. nov.; however, the latter has unremarkable fore tarsomeres 2 and 3. Peculiarly, Syrovátka [20] compared this species with *E. florisomna* Loew, 1856, despite the latter being quite different (with multiserial acrostichals, bare hypandrium, etc.). Females are rather similar to *E. morosa*; differences are given in the key.

**Distribution**: Switzerland, Austria. **Dates**: vi.–viii.

**

**Key to West Palaearctic species of *Empis* (*Empis*) *chioptera* group**

**

**Males**

**

The species *Rhamphomyia galactoptera* (recently also treated as *Empis*) is formally included due to similar male genitalia.

**


1 Vein R_4+5_ not forked. .......................................................................................
***Rhamphomyia galactoptera* Strobl, 1893**
- Vein R_4+5_ forked. .................................................................... 22 (1) Hypandrium setose. ........................................................ 3- Hypandrium without setae. .................................................. 43 (2) Abdominal setae black. Halter black (Switzerland, Austria). ......................................................................................
***E. scaura* Loew, 1867**
- Abdominal setae yellow. Halter yellowish brown to brownish yellow. ......................................................................
***E. dasyprocta* Loew, 1867**
4 (2) All body setae whitish (Israel). .........................................
***E. leucotricha* Collin, 1960**
- At least some setae (acrostichals, dorsocentrals, scutellars) black. .................................................................................. 55 (4) Abdomen silvery in dorsal view. ............................................................................................... 6- Abdomen grey, brown to black in dorsal view. ................................................................................................. 76 (5) Acrostichals and dorsocentrals irregularly 4-6 serial. Occiput below neck with very long dense pubescence. ........
***E. florisomna* Loew, 1856**
- Acrostichals and dorsocentrals biserial. Occiput with unremarkable setation. ............................................................***E. beckeriana***  
**Engel, 1946**
7 (5) Abdomen with pale (white to yellow) setae. ......................................................................................................... 8- Abdomen with black setae. ....................................................... 148 (7) Postpronotum and notopleuron with black setae. Laterotergite usually with black setae or with pale setae intermixed. .................................................................................... 9- Postpronotum and notopleuron with black and pale setae. Laterotergite usually with pale setae. .................................................................................... 119 (8) Mesoscutum subshiny black. Hypandrium short, not prolonged dorsally. ...............................................................
***E. prodromus* Loew, 1867**
- Mesoscutum grey microtrichose. Hypandrium long, prolonged dorsally. .................................................................................... 1010 (9) Seventh abdominal segment with very short hind marginal setae. Epandrium without dense ventral setae (Spain, Sicily). ...............................................................
***E. sicula* Loew, 1867**
- Seventh abdominal segment with long hind marginal setae. Epandrium with dense ventral setae. ....................................................................................
***E. pseudoprodromus* Collin, 1969**
11 (8) Tarsi of fore and mid legs and mid tibiae with very long brownish yellow hair-like setae (Caucasus). ...............................................................
***E. abagoensis* Kustov & Shamshev, 2013**
Fore and mid leg with usual setae. .................................................................................... 1212 (11) Anal vein incomplete. Stylus including first segment about as long as postpedicel (Israel). ...............................................................
***E. curticornis* Collin, 1960**
- Anal vein complete. Stylus shorter than postpedicel. ............................................................... 1313 (12) Stylus one third as long as postpedicel. Acrostichals black. First fore tarsomere broader than first hind tarsomere. Hypandrium elongate at apex. ....................................................................................
***E. chioptera* Meigen, 1804**
- Stylus three quarters as long as postpedicel. Anterior acrostichals pale. First fore tarsomere narrower than first hind tarsomere. Hypandrium short. (Caucasus). ....................................................................................
***E. pseudochioptera* Kustov & Shamshev, 2013**
14 (7) Anal vein incomplete, either vanishing before wing margin or invisible in apical third (but extreme tip may be again apparent). .................................................................................... 15- Anal vein complete. ......................................................................................................... 2215 (14) Wing milky white, veins (except C, R) white. Hypopygium of *chioptera*-type (cerci L-shaped and higher than epandrium). Fore and hind first tarsomeres almost equally wide. .................................................................................... 16- Wing not milky white with all veins dark. Hypopygium of *aestiva* type (with cerci longer than high, armed with spines or spine-like outgrowths or tips apically or medially). Fore first tarsomere slightly narrower than hind one. .................................................................................... 1716 (15) Epandrium without long setae apically. Abdomen lustrous even dorsally (best visible on tergites 6–7). ...............................................................***E. pusio*** 
**Egger, 1860**
- Epandrium with long setae apically (Figure 1D). Abdomen microtrichose dorsally (best visible on tergites 6–7) (Portugal). .........................................................................................................
***E. lusitanica* sp. nov.**
17 (15) Epandrium with very long setae around tip both dorsally and ventrally (Figure 3B). Phallus tapering evenly, without thickenings (Figure 3C). Simultaneously, mid tibia with long apical seta (nearly as long as first tarsomere) and mid femur with sparse (about 10) anteroventral setae (Portugal). ...............................................................
***E. miroslavi* sp. nov.**
- Epandrium without conspicuously long setae around tip. Phallus with basal thickening. Remaining characters not in above combination. ............................................................... 1818 (17) Fore tibia with posterodorsal setae fine, homogeneous and very short, shorter than tibia depth. Mid femur with dense (more than 15 setae) nearly equally short anteroventral setae (at most with several somewhat longer setae subbasally). .................................................................................... 19- Fore tibia with posterodorsal setae longer than diameter of tibia, not homogeneous. Mid femur with anteroventral setae sparse or, if dense, not equally long. .................................................................................... 2019 (18) Mid tibia with preapical seta much shorter than ventral setae. Mid first tarsomere with short apical setae. Hypandrium with upcurved tip narrower than narrow part of phallus. Cercus without anterodorsal ornamental spines. Phallus with narrow apical part about as long as slightly swollen basal part (see [2], Figs 214–217). ......................................................................................................***E. aestiva*** 
**Loew, 1867**
- Mid tibia with preapical seta much longer than ventral setae. Mid first tarsomere with long apical seta (nearly as long as next tarsomere). Hypandrium with upcurved tip broader than narrow part of phallus. Cercus with a row of anterodorsal ornamental spines. Phallus with narrow apical part scarcely one fourth as long as strongly swollen median part (Figure 2A–D) (Portugal, Spain). .......................................................................................................***E. manteigasensis* sp. nov**.20 (18) Mid femur with anteroventral setae sparse, longer in basal third than apically. Hypandrium with dorsally bent tip long, V-shaped forked apically. Cercus apically with two long spine-like outgrowths (see fig. 220 in [2]). Mid first tarsomere with long apical seta. Usually 2 scutellar setae. ........................................................................................
***E. praevia* Collin, 1927**
- Mid femur with anteroventral setae longer on apical third. Hypandrium short or long, not apically forked. Cercus apically without long spine-like outgrowth or with single one. Mid first tarsomere with short apical seta. Total of 2–4 scutellar setae. ................................................................................................ 2121 (20) Usually 4 scutellar setae. Cercus without conspicuous internal spine-like setae. Hypandrium very short, its dorsally bent part shorter than diameter of phallus. Phallus broadened in basal third with tip bent caudad (see figs 223–226 in [2]). ....................................................***E. levis*** **Loew, 1873**- Total of 2 scutellar setae. Cercus with about 30 internal spine-like setae. Hypandrium longer, its dorsally bent part much longer than diameter of phallus. Phallus broadened almost throughout its length with tip bent craniad (Figure 5B–E). (Spain). ................................................................................................
***E. serviae* sp. nov.**
22 (14) Epandrium with a single very long apical seta much longer than other setae. ........................................................................................ 23- Epandrium without conspicuously long apical seta. .............................................................................................................. 2423 (22) Epandrium acute at apex, without long setae dorsally of long apical seta (see figs 1–2 in [21]). Mid tibia usually with anterodorsal setae three times longer than its diameter (Switzerland, Austria, Romania). .............................................................................
***E. mariae* Syrovátka, 1991**
- Epandrium rounded at apex, dorsally of long seta with setae about half as long as this (see fig. 211 in [2]). Mid tibia usually with anterodorsal setae scarcely twice as long as its diameter, preapical similarly short. ..................................................................
***E. caudatula* Loew, 1867**
24 (22) Epandrium with a tuft of long setae. ....................................................................... 25- Epandrium not densely and long setose (If fore tarsomeres 2 and 3 distinctly swollen and densely setose, compare ***E. scaura***). ............................................ 2625 (24) Eighth sternite lustrous contrastingly to preceding ones. Four scutellar setae (Bulgaria). .......................................................***E. moceki***  
**sp. nov.**
- Eighth sternite microtrichose as preceding ones. Two scutellar setae (Israel). .......................................................
***E. cilicauda* Collin, 1960**
26 (24) Hypandrium depigmented ventrally, with sudden constriction in apical part (best apparent in ventral view) (Figure 7A). M veins brownish yellow to yellowish brown. Mesoscutum rather light brownish grey with somewhat darker and rather brownish stripes along rows of setae. Acrostichals shorter (about 0.10 mm) and more numerous (about 15 in one row) (Czech Republic, France, Switzerland.). .......................................................................
***E. morosa* Meigen, 1822**
- Hypandrium equally narrowing towards tip. M veins white (if R_4+5_ vein brown, compare ***E. pusio***). Mesoscutum deep black, subshiny, without stripes. Acrostichals longer (about 0.15 mm) and less numerous (less than 10 in one row). ................................. 2727 (26) Four scutellars. Wing more than 3.6 mm long. Fore femur with very long posteroventral setae, these in apical third about twice as long as femur depth. Fore tarsi with long apical setae. Abdominal sternites short setose. Dorsoapical angle of lower cercal lobe rectangular. Hypandrium longer. Phallus simply bowed preapically. (If hypandrium short and preapical anterodorsal seta on mid tibia short, compare ***E. caudatula*** with occasionally broken large seta on epandrium) (Bulgaria, Slovakia, Italy). ....................................................
***B. troyanensis* sp. nov.**
- Two scutellars. Wing less than 2.7 mm long. Fore femur with shorter posteroventral setae. Fore tarsi with short apical setae. Abdominal sternites long setose. Dorsoapical angle of lower cercal lobe rounded. Hypandrium shorter. Phallus S-shaped, curved preapically (Caucasus). (Aberrant specimens of *Rhamphomia galactoptera* lead to this point; they have 3 anterodorsal setae on mid tibia and one single very long ventral seta on hind femur). .........................................................***E. kamyshanovensis***  
**Kustov & Shamshev, 2013**



**

**Females**

It is difficult to arrange females into species groups, and, therefore, this key is only preliminary. Several species described only according to female sex traits may belong to the *chioptera* group too; however, current knowledge does not allow us to arrange them precisely within the key. *Empis surata* Kuntze is poorly known, and based only on the original description, the holotype female was not found in the Dresden Museum; this leads to a couplet with *E. beckeriana*; however, it should have broadened hind tibiae and tarsi. Type locality: Kotor (= Cottora) in Montenegro. *Empis helophila* Loew, 1867, was redescribed by Syrovátka [20], including type information and lectotype (female) selection. The following key leads to *E. morosa*, which may be identical (the female of *E. morosa* was unknown to Syrovátka). *Empis corvina* Loew, 1869, was redescribed by Syrovátka [20], including type information and lectotype (female) selection. In the following key, it leads to *E. morosa*; however, the first tarsomere of the hind leg may bear several flattened setae dorsally. *Empis fumosa* Loew, 1867, and *E. hirta* Loew, 1865, are known only based on females, and their affiliation with the *E. chioptera* group is doubtful.

**


1 Labrum at least 2.5x longer than head height. ................................ species other than *chioptera* group.- Labrum at most 2x longer than head height. ...................................... 22 (1) Abdominal setae at least partly (sides of first segment) white. (Unknown female of ***E. leucotricha*** from Israel may belong here: all body setae probably whitish. Unknown female of ***E. curticornis*** from Israel may belong here: may have incomplete anal vein. Unknown female of ***E. abagoensis*** from Caucasus may belong here. ......................................................................................................... 3- Abdominal setae completely black. ......................................................... 93 (2) Legs without flattened setae. ...................................................................................................... species other than *chioptera* group.- Legs with pennate or at least distinctly flattened setae. ........................................................................................................ 44 (3) Postpronotum and anterior part of notopleuron at least partly with white setae. ........................................................................................................ 5- Postpronotum and anterior part of notopleuron with only black setae. ............................................................................................. 65 (4) Antenna with stylus 1/3 of postpedicel length. Acrostichal and dorsocentral setae short. Acrostichal setae black throughout. ............................................................................................
***E. chioptera* Meigen, 1804**
- Antenna with stylus 3/4 of postpedicel length. Acrostichal and dorsocentral setae long. Acrostichal setae pale on anterior part of scutum. ............................................................................................
***E. pseudochioptera* Kustov & Shamshev, 2013**
6 (4) Mesoscutum subshining, with distinct shine. Hind femur convex and fringed with flattened setae dorsally and nearly straight and not pennate ventrally. ..............................................................................
***E. prodromus* Loew, 1867**
- Mesoscutum microtrichose, without distinct shine. Hind femur pennate ventrally. ............................................................................... 77 (6) Halter brownish. Sternites 2–3(4) lustrous. Hind tibia with homogeneous ciliation dorsally, without longer setae. ....................................................................................................................
***E. dasyprocta* Loew, 1867**
- Halter black. Other characters different. .................................................................................................. 88 (7) Middle femur without pennation. ........................................................
***E. sicula* Loew, 1867**
- Middle femur pennate (if wing brownish and coxae with pale setae, compare ***E. chioptera***). ......................................................................
***E. pseudoprodromus* Collin, 1969**
9 (2) Anal vein incomplete or indistinct before margin and again with short stub apparent at wing margin (unknown females of ***E. miroslavi*** sp. nov. from Portugal, ***E. manteigasensis* sp. nov.** from Portugal and Spain, and ***E. serviae*** sp. nov. from Spain would probably run here). .................................................................................... 10- Anal vein complete (even if white, traceable along all length to wing margin) (unknown females of ***E. cilicauda*** and ***E. moceki*** may belong here). ................................................................................. 1510 (9) Mid and hind femora with long pennation ventrally. Fore tibia without pennate setae dorsally. ......................................................
***E. aestiva* Loew, 1867**
- Not as above. .......................................................................................... 1111 (10) Hind femur convex and pennate dorsally, straight and nearly bare ventrally. ................................................................................... 12- Hind femur without flattened setae. ....................................................................................................... 1312 (11) Both fore and hind tibiae without pennate setae dorsally. ..........................................................................................
***E. pusio* Egger, 1860**
- Both fore and hind tibiae with pennate setae dorsally. ...........................
***E. lusitanica* sp. nov.**
13 (11) Mid and hind femora and hind tibia practically bare. Usually 4 scutellar setae. ................................................................................
***E. levis* Loew, 1873**
- Mid femur anterodorsally with setae as long as its depth. Hind femur dorsally with pubescence at least as long as its diameter. Usually 2 scutellars. .................................................................................................... 1414 (13). Hind femur with anterodorsal setae in apical third up to as long as its diameter. Hind first tarsomere with a single long posterodorsal seta. ....................................................................................................
***E. praevia* Collin, 1927**
- Hind femur without anterodorsal setae. Hind first tarsomere without long posterodorsal seta. (Not member of *chioptera* group). .................... [***E. dalmatica***]15 (9) Hind femur convex dorsally, pennate dorsally and ventrally. Front tibia and hind femur not pennate. Mid tibia short and rather thick. A total of 4 scutellar setae. ............................................................
***E. caudatula* Loew, 1867**
- Different characters. ................................................................................ 1616 (15) Acrostichals irregularly 4–6 serial. (If prosternum setose, compare ***E. nigricoma. E. fumosa*** and ***E. hirta*** probably also belong here; however, prosternum setosity unknown. Both may have 8–12 scutellars.)....................................................................................................
***E. florisomna* Loew, 1856**
- Acrostichals biserial. ................................................................................ 1717 (16) Front tibia and first tarsomere dorsally with pennate (or at least distinctly flattened) setae. ........................................................................... 18- Front tibia and first tarsomere not pennate dorsally. ................................................................................................... 2018 (17) Hind tibia ventrally in basal half broadly pennate. .............................................................................................. species other than *chioptera* group.- Hind tibia ventrally not pennate, at most with several slightly flattened setae. .................................................................................................... 1919 (18) Mid femur dorsally with slightly flattened setae. Wing brown. .............................................................................
***E. morosa* Meigen, 1822**
- Mid femur dorsally without pennation. Wing greyish, more distinctly so in anteroapical part. ................................................................
***E. scaura* Loew, 1867**
20 (17) Wing and calypter clear, almost milky white (mid and hind femora without flattened setae). ...............................................................................................
***E. beckeriana* Engel, 1946**
- Wing brownish. Calypter brown. ............................................................................ 2121 (20) Total of 2 scutellars. Mid and hind femora with fringes of flattened setae both dorsally and ventrally (aberrant specimens of *Rhamphomyia galactoptera* lead to this point; however, they have femora without flattened setae). ............................................................................
***E. kamyshanovensis* Kustov & Shamshev, 2013**
- Total of 4 scutellars (outer pair short). At least mid femur without flattened setae (if mid femur pennate on both sides, compare ***E. caudatula***). ............................................................................................... 2222 (21) Mesoscutum sparsely microtrichose, sublustrous in dorsal view. Hind femur ventrally almost bare on basal half, apically with anteroventral setae as long or longer than femur depth, sometimes setae with tendency to be slightly flattened. .........................................................
***E. troyanensis* sp. nov.**
- Mesoscutum rather densely microtrichose, light grey in dorsal view. Hind femur ventrally almost equally setose, apical setae not much longer than basal, no tendency to be flattened. ..................................................................................................................
***E. mariae* Syrovátka, 1991**



**

## Data Availability

The original contributions presented in this study are included in the article. Further inquiries can be directed to the author.
